# Vehicle Re-Identification Method Based on Efficient Self-Attention CNN-Transformer and Multi-Task Learning Optimization

**DOI:** 10.3390/s25102977

**Published:** 2025-05-08

**Authors:** Yu Wang, Rui Li, Yihan Shao

**Affiliations:** 1 College of Computer Science and Technology, Jilin University, Changchun 130012, China; ruili22@mails.jlu.edu.cn; 2 College of Software, Jilin University, Changchun 130012, China; shaoyh5524@mails.jlu.edu.cn

**Keywords:** vehicle re-identification, intra-class variations, inter-class similarities, self-attention mechanism, multi-branch architecture, multi-task learning

## Abstract

To address the challenges of low accuracy in vehicle re-identification caused by intra-class variations, inter-class similarities and environmental factors, this paper proposes a CNN-Transformer architecture (IBNT-Net) for vehicle re-identification. The method builds upon a ResNet50-IBN backbone network and incorporates an improved multi-head self-attention mechanism to aggregate contextual information. It constructs a multi-branch vehicle re-identification network that combines both global and local features. Furthermore, a multi-task learning strategy is adopted, creating specialized learning pathways for classification tasks and metric learning tasks. Group convolution techniques are utilized to reduce model complexity, making it suitable for resource-constrained environments. On the VeRi-776 and VehicleID dataset, the proposed method achieves state-of-the-art performance with less parameters. The experimental results show that the proposed method has better re-identification performance and the extracted features are more discriminative.

## 1. Introduction

Vehicle re-identification is a technique for determining the presence of a particular vehicle in an image or video using computer vision techniques, i.e., given a vehicle image, retrieving the image of that vehicle from different camera views. Vehicle re-identification has a broad application prospect in the fields of intelligent transportation, intelligent security, and criminal investigation. However, due to the different camera shooting viewpoints and the interference of the environment where the vehicles are located, such as weather, shading and other factors, the same vehicle presents a very different appearance in different viewpoints, while non-identical vehicles may be extremely similar in appearance. This intra-class variability and inter-class similarity significantly increases the learning difficulty of vehicle discriminative features, posing a great challenge for the vehicle re-identification task.

Vehicle re-identification and sensor technology exhibit a bidirectional driving relationship. Sensor technology, through multi-view camera configurations and high-resolution imaging devices, provides algorithms with multidimensional data inputs encompassing vehicle appearance details, pose variations, and motion characteristics. For instance, multi-view data mitigate intra-class variations under single-view conditions, while high-resolution images enable precise extraction of localized key features. Conversely, the demand for feature discriminability and generalization capability in vehicle re-identification algorithms drives the optimization of sensor configurations toward higher resolution, broader view coverage, and dynamic capture capabilities. This synergistic evolution not only enhances vehicle matching accuracy in complex lighting and occlusion scenarios but also provides technological support for real-time perception and data-driven decision-making in intelligent transportation systems.

## 2. Related Work

Early vehicle re-identification research [1,2,3] was mainly based on sensors or artificially designed features, which had some discriminative ability but had significant limitations, mainly in terms of dependence on sensors, sensitivity to external conditions, complexity of feature engineering, and high deployment costs. In recent years, the field of computer vision has experienced rapid development, and deep learning-based methods have gradually become the mainstream direction of vehicle re-identification research, which can be categorized into Convolutional Neural Networks-based vehicle re-identification methods. Transformer-based vehicle re-identification methods, based on the different architectures and feature extraction mechanisms.

Convolutional neural networks extract local features of an image through multi-layer convolutional operations and gradually aggregate them into global features. Liu et al. [4] proposed a deep relative distance learning method, which utilizes a two-branch deep convolutional network to project the original vehicle images into a Euclidean space, where the distance can be directly used to measure the similarity of any two vehicles. Rao et al. [5] proposed a counterfactual attention learning method based on causal inference, which can be widely used in different fine-grained visual identification tasks by adding an attention module to ResNet50 [6] for counterfactual intervention and attention learning. Ghosh et al. [7] proposed a relation-preserving ternary mining method, which considers that different viewpoints of an object (e.g., front, side, and rear views of a vehicle) form distinct natural subgroups, and the relationships among these subgroups should be maintained during the learning process. This is achieved by identifying triads through determining positive and negative samples that have a natural relationship with the target image to ensure viewpoint consistency. Gu et al. [8] proposed a novel dual comparison mechanism and multi-scale interactive search space to improve the performance of Neural Architecture Search (NAS) in heavy identification tasks. Li et al. [9] addressed the day-night cross-domain vehicle re-identification (DN-ReID) problem by proposing the Day-Night Dual-domain Modulation (DNDM) framework, which integrates glare suppression, structure enhancement, and class awareness to dynamically modulate day-night cross-domain vehicle features. They also introduced two benchmark datasets, DN-Wild and DN-348, to facilitate research in this domain.

The Transformer-based vehicle re-identification method captures long-range dependencies through global modeling with a self-attention mechanism. He et al. [10] proposed a Transformer-based object re-identification framework, which generates more robust and discriminative feature representations by introducing shifting and shuffling in the last layer of the Transformer. To address the issue of feature deviation caused by changes in viewpoint, an edge information embedding module is designed to incorporate non-visual cues, such as camera number and viewing angle, into the feature representation. Li et al. [11] proposed an object re-identification method based on CLIP, which solves the problem of the lack of specific textual labels in the re-identification task by leveraging the cross-modal descriptive capability of CLIP networks.

In recent years, many researchers have combined the advantages of convolutional neural networks in local feature extraction with those of Transformer in global information modeling and proposed a hybrid CNN-Transformer architecture, which has achieved impressive results in vision tasks, which not only improves the performance of the network in a variety of computer vision tasks but also achieves a better latency trade-off between latency and accuracy. Srinivas et al. [12] proposed a hybrid network (BoTNet) combining convolution and self-attention mechanisms, which significantly improves the performance of vision tasks by introducing self-attention mechanisms in the last three bottleneck blocks in ResNet.

In this paper, we draw upon the design concept of BoTNet and introduce the improved multi-head self-attention mechanism into the ResNet50-IBN [13] network, constructing a multi-branch architecture network that integrates global and local features; we place cross-entropy loss and triplet loss in independent branch optimization to reduce gradient conflicts and promote feature reuse; and we use a group convolution strategy to further optimize the network to achieve a balance between accuracy and efficiency.

The contributions of this paper are as follows:

1. A vehicle re-identification method based on the new double-branch CNN-Transformer architecture is proposed, which introduces the self-attention mechanism into the ResNet50-IBN backbone network to aggregate contextual information and constructs a multi-branch vehicle re-identification network combining global and local features.

2. A more efficient self-attention mechanism is proposed, which improves the computational efficiency and enhances the ability of global information aggregation compared with the original MHSA.

3. A multi-task learning strategy is used to optimize the cross-entropy loss and triplet loss by placing them in independent branches to reduce gradient conflicts and promote feature reuse.

4. The multi-branch network architecture is further optimized using group convolution techniques to improve computational efficiency and reduce complexity while maintaining performance advantages to meet the demands of real-time processing and efficient deployment in resource-constrained environments.

The remainder of this paper is organized as follows: Section 2 provides a detailed introduction to the related techniques and theoretical foundations employed in this study. Section 3 provides a detailed introduction to the proposed CNN-Transformer based vehicle re-identification method, as well as its detailed components in this study. Section 4 elaborates on the structure of the vehicle re-identification method based on multi-task learning optimization. Section 5 offers an in-depth introduction to the two datasets, explains the experimental implementation details, and presents the experimental data and visualized results. Section 6 concludes our research.

## 3. CNN-Transformer Based Vehicle Re-Identification Method

### 3.1. Overall Structure

**Figure 1:**
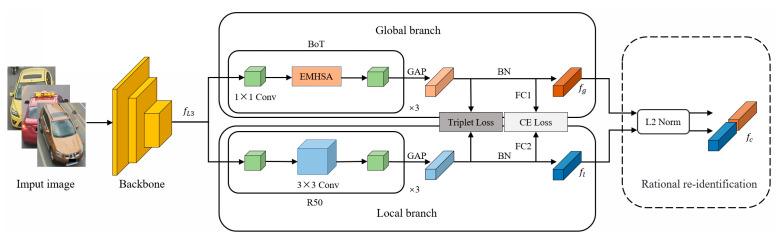
Architecture of IBNT-Net based on CNN-Transformer.

The IBNT-Net based on the hybrid CNN-Transformer architecture is shown in Figure 1. Vehicle images are extracted from the initial features by the backbone network ResNet50-IBN, and the invariant features about the target are learned by cleverly combining instance normalization and batch normalization in a deep network, which improves the modeling and generalization ability without increasing the network complexity. The global branching adopts the BoTNet structure, which is based on the Efficient Multi-Head Self-Attention (EMHSA) mechanism to capture long-range dependencies, and aggregates the global information.

The local branch uses the last set of residual blocks of ResNet50-IBN to further capture the advanced semantic information of the image. The features extracted from the global and local branches are stitched together by L2 normalization to form the complete features of the vehicle image. The combination of Label-Smoothed Cross-Entropy Loss and Triplet Loss helps the network to not only learn efficiently in the classification task but also achieve better performance in metric learning, resulting in a better distance metric in the feature space.

### 3.2. Backbone Network

**Figure 2:**
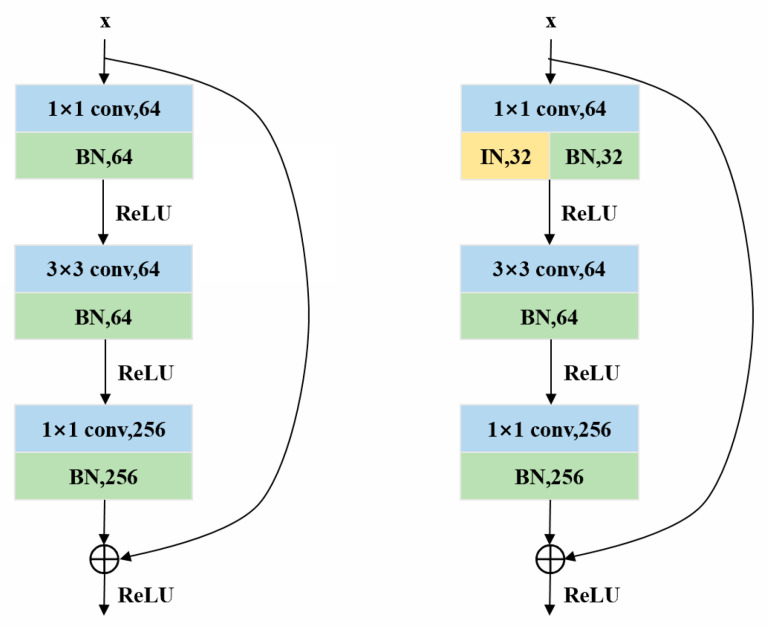
Residual block of ResNet50 and ResNet50-IBN.

As shown in Figure 2, the ResNet50 network makes extensive use of a batch normalization layer, which normalizes the output by calculating the mean and variance of the current batch to accelerate training and stabilize the training process. However, the batch normalization layer is affected by the appearance (e.g., illumination, change of viewing angle) and the difference of data distribution, which will lead to the degradation of the network’s generalization ability. In this paper, the backbone network adopts the ResNet50-IBN network structure, which uses both instance normalization and batch normalization in the shallow layer of the network, and only batch normalization in the deep layer on the network.

In the shallow network, appearance differences have a greater impact on features, and using instance normalization to perform a separate normalization operation for each sample instance allows the network to focus more on learning content features between samples, rather than being distracted by appearance differences. Batch normalization is used to stabilize the training process, reduce internal covariate bias, and help the network learn a stable feature representation faster. In deep networks, the feature differences brought about by appearance differences are already very small, when the differences between contents become the dominant factor, and batch normalization can help the network better capture these content features. Combining the above two normalization methods, the ResNet50-IBN backbone network is constructed by replacing half of the channels of the first normalization layer of the first three residual blocks of the ResNet50 network with the instance normalization layer, and the last residual block remains unchanged.

### 3.3. Two-Branch Feature Representation Network

**Figure 3:**
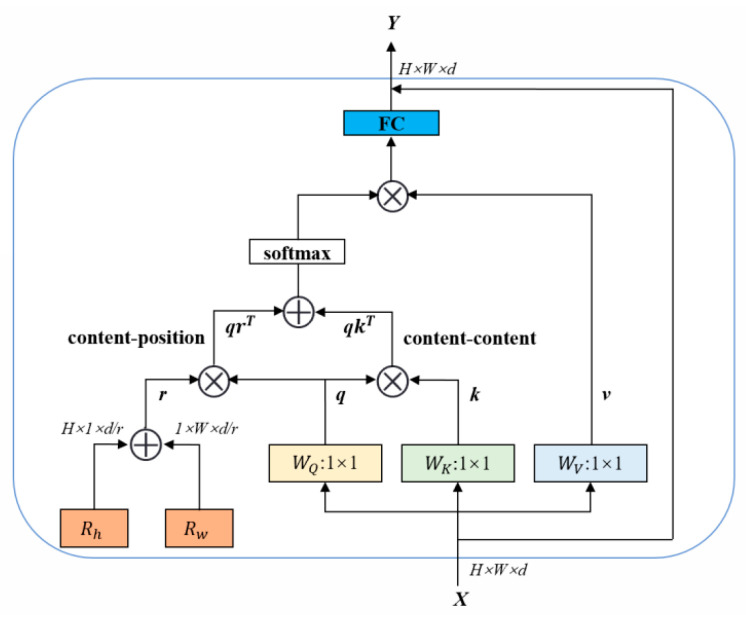
The EMHSA used in the global branch.

Initial features extracted through the backbone network can capture some discriminative semantic information, but it is difficult to fully express the global context information and local detail information in the image. In order to further enhance the feature representation ability of the network and fully extract the complete features of the vehicle image, a two-branch feature extraction network is constructed by integrating the global and local features, which extracts the global and local features of the vehicle image and splices them together to form the complete features. The local branch directly uses the last set of residual blocks of ResNet50-IBN, focusing on capturing higher-level abstract information and local detailed features in vehicle images, which usually contain rich texture, shape, and structure information and are important for distinguishing different vehicles. In the global branch, the 3 × 3 convolutional layer in the three bottleneck blocks of the last layer of ResNet 50-ibn is replaced by an efficient multi-head self-attention layer (EMHSA), which enables the network to better focus on the correlation between different regions in the image, thus effectively aggregating global context information and enhancing the network’s ability to capture long-distance dependencies. The structure of the EMHSA is shown in Figure 3.

EMHSA introduces a combination of reduction factor and Fully Connected Layer (FC) plus residual connectivity based on Multi-Head Self-Attention (MHSA) to improve computational efficiency and network performance. Specifically, the EMHSA layer uses four attentional heads to enhance the network’s ability to capture positional information by encoding the relative position R_h_ and R_w_ along the height and width directions, respectively.

When calculating Query(*q*), Key(*k*), and relative position encoding *r*, each is first divided by a reduction factor to reduce computational complexity (the computational complexity of attention weight calculation is reduced from *O*(*L*^2^*d*) to *O*(*L*^2^*d/r*), where *L* represents the total spatial dimension length of the feature map, and *d* denotes the number of channels) and control numerical stability. The attention score consists of the dot product *qk^T^* between *q* and *k* and the dot product *qr^T^* between *q* and *r*. The total attention score is denoted as *qk^T^* + *qr^T^*. The operator “⊕” denotes element-by-element addition, “⊗” denotes matrix multiplication, and “1 × 1” denotes 1 × 1 convolution for changing the number of channels without changing the feature map size. Next, the attention scores are normalized by the Softmax function to obtain the attention weight matrix, and matrix multiplication is performed with the Value (*v*) vector to generate the weighted summed feature representation. To further enhance the network representation, the generated feature representation is transformed through a fully connected layer, and, finally, the transformed features are residually connected to the input features to facilitate gradient flow and avoid information loss.

### 3.4. Loss Function

In the vehicle re-identification task, the goal of the network is to be able to accurately recognize the same vehicle captured by different cameras, even though these images may come from different viewpoints, lighting conditions, or points in time. To achieve this goal, the network needs to learn vehicle feature representations that are both discriminative and well-clustered in the feature space. However, it is often difficult for a single type of loss function to fulfill both requirements. Therefore, in this paper, we use a multi-loss function strategy that combines cross-entropy loss and triplet loss in order to fully take into account the needs of both the classification task and metric learning.

For the classification task, after extracting the embedding vectors, the vectors are reduced to the number of classes in training by a fully connected layer. Next, the Softmax function is applied, and the cross-entropy loss with label smoothing is computed according to the definition of reference [14], which is expressed as follows:

(1) $$L_{\mathrm{cls}}=-\sum_{c=1}^{C}y_{\mathrm{gt}}^{i}\log(y_{p}^{i})$$

where *C* is the number of categories in training, *y^i^_p_* is the predicted value of the probability that the target belongs to the category *i*, *y^i^_gt_* is the true label of the *i*-th category, *y^i^_gt_* = 0 denotes negative samples, and *y^i^_gt_* = 1 denotes positive samples. The formula for label smoothing is as follows:

(2) $$y_{\mathrm{gt}}^{i}=\left\{\begin{array}{ll} 1-\frac{\left(C-1\right)}{C}\epsilon & \mathrm{if}\;i=\mathrm{class},\\ \frac{\epsilon }{C} & \mathrm{if}\;i\neq \mathrm{class}. \end{array}\right.$$

where ϵ is the smoothing coefficient, which reduces the confidence of the network prediction by label smoothing, prevents the network from overfitting, and improves the robustness of the network.

For metric learning, a ternary loss [15] is used, and training batches are constructed using randomized PK sampling with P identities each, and K samples each. Each image is used as a pinpoint, and the “hardest” sample is obtained; the hardest positive sample is the image with the largest distance from the pinpoint among the images with the same identity as the pinpoint, while the hardest negative sample is the image closest to the anchor among the images with different identities than the anchor. This loss function expression is given as follows:

(3) $$L_{tri}=\frac{1}{PK}\sum_{i=1}^{PK}\max\left(\begin{array}{l} 0,m+\max_{p\in P(a)}[d(x_{a},x_{p})]\\ -\min_{n\in N(a)}[d(x_{a},x_{n})] \end{array}\right)$$

where P(a), N(a), and m denote the set of positive samples, the set of negative samples, and the distance bounding threshold of the pinpoints; *x_a_*, *x_p_*, and *x_n_* denote the extracted embedding of the pinpoints, the positive samples, and the negative samples, respectively; and *d*() denotes the Euclidean distance.

Finally, the cross-entropy loss and the triplet loss are summed to obtain the final loss. This setup ensures that the computation of the loss function takes into account the needs of both classification tasks and metric learning, allowing the network to be better optimized for training.

## 4. Vehicle Re-Identification Method Based on Multi-Task Learning Optimization

Multi-task learning is an important strategy in the field of deep learning, the core idea of which is to train multiple related tasks simultaneously in a network, where multiple tasks share the underlying feature extractor, and each task owns an independent branch or module, and the correlation between tasks is utilized to achieve information and feature representation sharing. For vehicle re-identification, both the classification task and the metric learning task are essentially designed to improve the effectiveness of vehicle re-identification.

Although these two tasks are not completely independent tasks, there are significant differences in their technical objectives and optimization approaches. Specifically, the classification task (cross-entropy loss) aims to maximize inter-class feature differences for discriminative purposes, while the metric learning task (triplet loss) focuses on optimizing the geometry of the feature space to enhance intra-class compactness and inter-class separability. The gradient optimization directions of these two tasks may conflict: for instance, classification might push features of different vehicles toward distinct class centers, whereas metric learning requires features of the same vehicle to cluster tightly in the space, potentially causing conflicting gradient directions in the shared network layers. Therefore, from the perspective of technical implementation, multi-task learning can be adopted in the form of placing different loss functions in separate branches for optimization to promote the synergy effect between classification and metric learning and avoid gradient conflicts. This design can not only effectively solve the problems existing in the traditional joint optimization method but also give full play to the advantages of the two tasks to further improve the network performance. In addition, in order to solve the problem of network complexity brought by the multi-branch architecture, while trying to maintain the network performance as much as possible to meet the requirements of real-time processing and efficient deployment in resource-constrained environments, the group convolution strategy is introduced to optimize the network.

### 4.1. Multi-Branch Feature Extraction Network Based on Multi-Task Learning

For the IBNT-Net proposed in the previous section, the global and local branches are still distinguished, based on which, the cross-entropy loss and triplet loss are placed in independent branches for optimization in order to reduce the gradient conflict and promote the feature reuse, and, finally, a re-identification network with a four-branch architecture is obtained. The branches of the architecture share weights until the last set of residual blocks of the backbone network, and the final features of each branch are generated in the following way:

(4) $$f_{N}(x)=GAP[F_{N4}(F_{s123}(x;\theta_{s});\theta_{N})]$$

where *f_N_* denotes the feature embedding of the *N*-th branch, *x* is the input image, *F_N_*_4_ denotes the *N*-th branching layer, *F_s_*_123_ denotes the backbone network in front of the multi-branch network, *GAP* denotes the global average pooling, $\theta_{s}$ is the shared weights, and $\theta_{N}$ is the weight of the first *N* branch. The embeddings obtained from each branch are L2 normalized to ensure equivalent contributions and then stitched together to obtain the final feature representation. In the above way, the multi-branch architecture extracts multiple global embedding, and each branch has a specific task and feature extraction method, focuses on learning different types of information, and, finally, obtains diversified features with richer information. When the network is trained, each branch loss is trained independently, and the global loss is obtained by a weighted linear combination of each branch loss as follows:

(5) $$L=\sum_{i=1}^{N}\omega_{cls}^{i}L_{cls}^{i}+\omega_{tri}^{i}L_{tri}^{i},\left\{\begin{array}{cc} \omega_{tri}^{i}=0 & \mathrm{if}\;\mathrm{classification}\;\mathrm{branches}\;\\ \omega_{cls}^{i}=0 & \mathrm{if}\;\mathrm{metric}\;\mathrm{learning}\;\mathrm{branches}\; \end{array}\right.$$

where *N* denotes the number of branches; for categorization branches, only categorization loss is computed, and the weight of metric loss is set to 0. For metric branches, only metric loss is computed, and the weight of categorization loss is set to 0. The loss weights of each branch $\omega^{i}$ are adjusted by cross-validation to balance the importance of different tasks and maximize the overall performance.

### 4.2. Group Convolution Strategy

**Figure 4:**
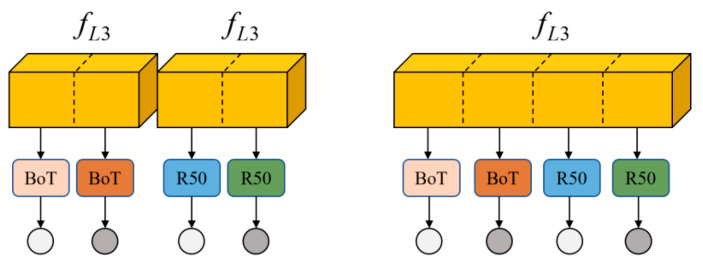
Architectural variants employing different group convolution strategies.

A variant of the architecture using different grouped convolution strategies is shown in Figure 4. The architecture is directly based on the image features initially extracted by the ResNet50-IBN backbone network for processing, with the BoT and R50 corresponding to the global and local branches in the multi-task learning vehicle re-identification network above, respectively, and the loss functions represented by circles, with the lighter color representing the cross-entropy loss and the darker color representing the ternary loss. The left figure shows that the features extracted from the backbone network are first expanded into two groups, and then the grouping convolution operation is applied to each group of features separately, which reduces the computational burden of each branch by grouping the features, while retaining sufficient feature expressiveness.

Compared with the architecture without grouped convolution, this method can reduce the number of parameters and computational complexity to some extent, while maintaining better network performance. On the right, the features extracted from the backbone network are directly divided into four groups, and each group performs the convolution operation independently. This design significantly reduces the number of parameters and computational cost of each branch, which makes the network more lightweight and suitable for deployment in resource-constrained environments.

## 5. Experimentation and Analysis

The experiments in this paper were carried out on a computer with Windows 11 installed on an Intel(R)Core(TM) i7-14700KF processor, 1 RTX 4070ti (12 GB) graphics card, and 64 G RAM. For the deep learning framework, the widely used PyTorch 2.3.0 framework is adopted. The development, debugging, and running of the code are performed in PyCharm. In order to facilitate environment management and dependency control, the Python environment was configured using the Anaconda tool. The network performance is evaluated on VeRi-776 [16] and VehicleID [4] datasets using the mean average precision (mAP) and the probability of correctly matching a target in the first N retrieval results (Rank-N). The dataset contains more than 50,000 images of 776 different automobiles, where the training set contains 37,778 images of 576 automobiles. The VehicleID dataset contains 221,763 images of 26,267 automobiles, and its test set is further divided into three subsets: large, medium, and small.

### 5.1. Experimental Setup

During the training phase, the vehicle images input to the network are uniformly adjusted to 256 × 256, and random cropping, level flipping, and random erasure data enhancement techniques are used to improve the generalization ability of the network. A randomized PK sampling method is used in the construction of the training batches to both include sufficient identity diversity and provide rich sample variations within the same identity. Setting P = 6, K = 8 for the VeRi-776 dataset and P = 16, K = 4 for the VehicleID dataset results in batch sizes of 48 and 64, respectively. All network layers, except the global branch, use weights pre-trained on ImageNet.

In order to align the global branches with randomly initialized weights with the pre-trained weights, a two-step fine-tuning method is used in this paper. At the initial training, only the global branch is trained, and the weights of the rest are kept frozen, using the Adam optimizer [17] and a fixed learning rate of 10^−4^ to align the randomly initialized weights of the global branch with the pre-training weights, to avoid unstable training due to the large difference in weights. After the initial training of the global branches, the whole network continues to be trained for 120 cycles, still using the Adam optimizer, with an initial learning rate of 10^−4^, linear warm-up in the first 10 cycles, and decreasing the learning rate by 0.1 at the 40th, 70th, and 100th cycles. A combination of cross-entropy loss and triplet loss is used to guide the training optimization of the network, where the label smoothing factor in cross-entropy loss ϵ=0.1, and the boundary threshold in triplet loss m = 0.1. For the vehicle re-identification network optimized based on multi-task learning, the classification loss weight is set to 0.6 and the metric loss weight is set to 1.0.

In the testing phase, the test set information of the trained network and dataset is loaded, and, for each query sample in the query set, calculate the similarity between it and each sample in the gallery set, sort the samples according to the similarity size, obtain the corresponding retrieval results, and calculate the mAP, Rank-1, and Rank-5 metrics based on the retrieval results.

### 5.2. Ablation Experiments

All the ablation experiments in this paper are performed on the VeRi-776 dataset. In order to analyze the effect of different structural designs on the network performance, the ablation experiments are conducted based on the ResNet50 network. The experimental results are shown in Table 1. By combining instance normalization and batch normalization to improve the residual blocks in the shallow layer of the ResNet50 network, the network mAP and Rank-1 are improved by 0.15% and 0.53%, respectively. ResNet50-IBN eliminates the style variations between different samples by performing the normalization operation on each sample instance separately through instance normalization and filters out the information reflecting the appearance variations while retaining the semantic information through the batch normalization to retain semantic information and better capture key features in the image.

By introducing a multi-head self-attention layer to replace the 3 × 3 convolutional layer in the last set of residual blocks of ResNet50-IBN, the BoT50-ibn network is obtained, and the mAP is further improved by 0.29%, but the Rank-1 is slightly decreased. This is due to the fact that the self-attention mechanism is able to capture long-range dependencies and enhance the aggregation of global information, but replacing the 3 × 3 convolutional layer leads to the loss of high-level semantic information. mAP and Rank-1 are different optimization objectives; mAP focuses on the average performance of the whole ranked list, while Rank-1 focuses on the accuracy of the first prediction, and different optimization objectives lead to different performance.

For the CNN-Transformer-based two-branch vehicle re-identification network IBNT-Net proposed in this paper, its mAP and Rank-1 are improved by 2.36% and 0.66%, respectively, indicating that the network not only inherits the inherent advantages of ResNet50-IBN but also makes the network able to capture the contextual and semantic information of the image at the same time through the introduction of a self-attention mechanism that further enhances the network’s characterization ability.

In order to verify the impact of different optimization strategies for self-attention mechanisms on network performance based on the IBNT-Net, a reduction factor and a combination of fully connected layers and residual connections were introduced into the original self-attention mechanism. The results are shown in Table 2. By only introducing the reduction coefficient, the mAP increased by 0.07%. The dimensionality reduction operation can reduce computational complexity, improve numerical stability, and avoid overemphasizing or neglecting certain positions.

By separately introducing the combination of fully connected layers and residual connections, the mAP and Rank-1 increased by 0.24% and 0.06%, respectively. The combined use of fully connected layers and residual connections enhances feature representation while preventing gradient vanishing/explosion or information loss, allowing the network to better retain and enhance useful information. After combining the two strategies to improve the self-attention mechanism, the mAP and Rank-1 increased by 0.57% and 0.18%, respectively, while Params and MACs decreased by 0.68 M and 0.18 G, respectively.

In order to verify the effect of the shrinkage coefficient on the network performance in the self-attention mechanism, based on the IBNT-Net, different shrinkage coefficients are set, and the results are shown in Table 3. When the shrinkage coefficient is 8, Rank-1 is slightly higher, but the mAP is lower. A smaller shrinkage coefficient makes the attention score larger, which will lead to the attention score of some positions being too high, while the score of other positions is too low, resulting in the network not being balanced enough in allocating the attention; when the shrinkage coefficient is 32, although the numerical stability is better, it lacks in the feature extraction, which leads to the loss of information, and the overall performance is slightly lower; when the shrinkage coefficient is 16, the network has the best overall performance, with a mAP of 83.03%. A moderate reduction factor achieves a better balance between numerical stability, computational complexity, attention intensity, regularization effect, and feature retention.

In order to verify the impact of the multi-task learning optimization strategy on the performance of the vehicle re-identification network, comparative experiments were conducted on several networks, and the results are shown in Table 4.

After the multi-task learning optimization strategy is used to place the cross-entropy loss and the triplet loss in separate branches for optimization, the number of network branches expands twice, and the mAP and Rank-1 evaluation indexes are both improved significantly. The significant performance improvement is mainly due to the fact that the cross-entropy loss focuses on the differentiation ability between classes, aiming to maximize the inter-class distance, while the triplet loss focuses on the optimization of inter-sample distance, aiming to minimize the distance of the similar samples and maximize the distance of the dissimilar samples, and, by splitting the loss function into independent branches, each branch can focus on its own specific task objective, which makes the optimization process more efficient and stabler.

Different group convolution strategies are applied to the input channels with multi-branch structure in the network, and their effects on the network complexity and performance are verified through experiments, and the experimental results are shown in Table 5, where DL (Decoupled Loss) indicates that the multi-loss function is split into independent branches for optimization, and 2B and 4B denote the two-branch and four-branch architectures, respectively. Further, 2G denotes that the features are expanded into two groups of operations and then group convolution, and 4G denotes dividing the input channels into four groups for convolution operations, respectively.

For the four-branch vehicle re-identification network IBNT-DL4B proposed in this section, which employs a multi-task learning strategy, two variants, IBNT-DL2G and IBNT-DL4G, were obtained using different group convolution strategies. Experimental results show that group convolution strategies significantly reduce the number of parameters and computational costs, though the mAP and Rank-1 metrics experience some decline, the overall performance still outperforms the two-branch network IBNT-Net proposed in the previous section. This performance degradation stems from the increased number of groups reducing the number of learnable feature channels per group, thereby decreasing model capacity. When the number of groups is excessive, the sparsity of feature interactions may lead to critical detail loss, especially in scenarios with limited training data or high task complexity, potentially causing underfitting risks. In practical deployment, the choice of group configuration requires balancing performance and efficiency: under strict resource constraints (e.g., edge devices), the 4G configuration (IBNT-DL4G) can be prioritized, whose parameter count is only 23% of the IBNT-DL4B network and reduces computational costs by 53%; under moderate resource conditions, the 2G configuration (IBNT-DL2G) can be adopted to balance precision and efficiency; in high-accuracy demand scenarios, the original multi-branch architecture (IBNT-DL4B) is used to achieve optimal performance (84.85% mAP) at the cost of a higher computational overhead. In summary, the group convolution strategy effectively alleviates the computational complexity brought by multi-branch architectures, and, while maintaining performance advantages, provides greater flexibility for practical deployment of the network.

### 5.3. Experiment Results and Analysis

In order to validate the effectiveness of the method in this paper, a comparison is made with other methods on the VeRi-776 dataset and the VehicleID dataset, and the results are shown in Table 6. For a fair comparison, none of the experimental data in the table uses additional information such as camera number, view angle, etc. to enhance the network performance, and no reordering technique is used to reorder the retrieval results. In addition, on the VehicleID dataset, only a small-scale test subset is used for comparison, and only two evaluation metrics, Rank-1 and Rank-5, are compared. From the experimental data in the table, it can be seen that the method in this paper achieves competitive performance compared with previous CNN-based and Transformer-based methods.

**Table 6:** Comparison of different methods on the VeRi-776 and VehicleID datasets.

Method		VeRi-776		VehicleID	
		mAP	R1	R1	R5
CNN	PGAN [18]	79.3	96.5	77.8	92.1
	SAN [19]	72.5	93.3	79.7	94.3
	FIDI [20]	77.6	95.7	78.5	91.9
	PVEN [21]	79.5	95.6	84.7	97.0
	SAVER [22]	79.6	96.4	79.9	95.2
	CFVMNet [23]	77.1	95.3	81.4	94.1
	CAL [5]	74.3	95.4	82.5	94.7
	CLIP-ReID [11]	80.3	96.8	85.2	97.1
Transformer	TransReID [10]	80.6	96.9	83.6	87.1
	DCAL [24]	80.2	96.9	—	—
	CLIP-ReID	83.3	97.4	85.3	97.6
The proposed	IBNT-Net	83.0	97.6	86.9	97.7
	IBNT-DL4G	83.2	97.4	—	—
	IBNT-DL4B	84.9	97.7	87.3	97.8

On the VeRi-776 dataset, the method achieves 84.9%mAP and 97.7% Rank-1, while, on the VehicleID dataset, it achieves 87.3% Rank-1 and 97.8% Rank-5, respectively. Even with the grouped convolutional strategy to reduce the complexity and improve the computational efficiency, the method still maintains competitive re-identification performance while maintaining competitive re-identification performance. This demonstrates the effectiveness of the group convolution strategy in balancing network performance and efficiency and also shows the potential of combining multi-task learning optimization strategies with group convolution.

**Figure 5:**
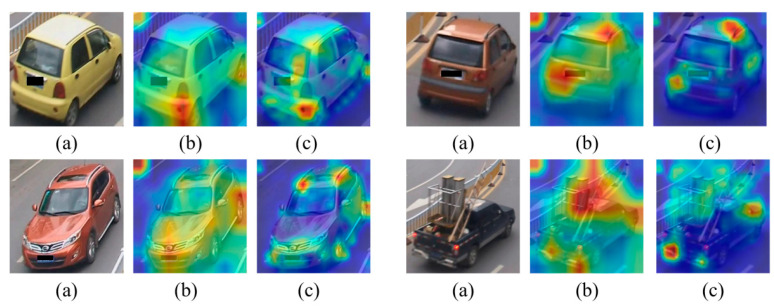
Visual comparison of feature map heatmaps: baseline ResNet-IBN vs. proposed IBNT-DL4B Network.

In order to study and explain the network behavior, the focus areas that the network focuses on when processing vehicle images are visualized by generating a heat map of the feature maps. Firstly, the feature maps output from the last layer of the model are obtained through forward propagation; then, the feature maps are subjected to an up-sampling operation to match their dimensions with the input image; finally, the maximum value is taken along the channel dimensions to generate a single-channel heat map, which is superimposed on top of the original image. As shown in Figure 5, (a) is the input vehicle image, (b) is the feature map heat map obtained from the baseline network ResNet50-IBN, and (c) is the feature map heat map obtained from the IBNT-DL4B network proposed in this paper.

As can be seen from the figure, the ResNet50-IBN network does capture some information about the vehicle features, but the attention is more dispersed, not only focusing on the critical parts of the vehicle but also giving more attention to the background region and the non-critical parts of the vehicle, and this dispersed attention may lead to the network’s performance limitation in distinguishing between different vehicles. In contrast, the method in this paper shows stronger focusing ability, which can effectively suppress the interference of background noise as well as non-critical parts of the vehicle, and focuses more on the key areas in the image that are easy to distinguish between different vehicles (e.g., luggage racks, lights, wheels, and bumpers, etc.), which is crucial for improving the accuracy of vehicle re-identification.

## 6. Conclusions

In this paper, we propose IBNT-Net, a vehicle re-identification method based on a hybrid CNN-Transformer architecture, enhanced through multi-task learning optimization and group convolution strategies. Experimental results demonstrate significant performance improvements on both the VeRi-776 and VehicleID datasets, particularly in key metrics such as mAP and Rank-1. Specifically, the best-performing variant, IBNT-DL4B, achieves 84.9% mAP and 97.7% Rank-1 accuracy on the VeRi-776 dataset. However, the current approach primarily relies on visual data (RGB images) for feature extraction, which, while effective in standard scenarios, limits its robustness in extreme conditions such as heavy rain, fog, or low-light environments where visual data may degrade. This limitation highlights the importance of integrating complementary sensor modalities to enhance generalization across diverse environments.

Future research will focus on two directions: first, deepening the application of multi-modal sensor fusion technology in vehicle re-identification tasks by developing a spatiotemporal alignment-based heterogeneous data fusion framework that integrates visual sensor images, LiDAR 3D point clouds, radar motion detection, and GPS spatiotemporal localization information. For instance, Huang X et al. [25] proposed L4DR, an end-to-end framework for LiDAR and 4D radar fusion, which addresses robustness issues in 3D object detection under adverse weather conditions. Secondly, a high-resolution, systematically designed large-scale multi-modal dataset will be constructed through synchronized multi-sensor data streams, integrating diverse environmental conditions. This dataset will incorporate heterogeneous sensor modalities including RGB images, LiDAR point clouds, radar signals, and GPS-based spatiotemporal metadata. (For instance, the Dual Radar dataset [26] synchronously captures multi-modal data from cameras, LiDAR, and two mainstream 4D millimeter-wave radars, covering complex urban traffic scenarios such as sunny days, rainy conditions, nighttime environments, and tunnels, demonstrating the feasibility of multi-sensor collaborative perception). The dataset will employ fine-grained annotations (e.g., occlusion levels of vehicle components, sensor noise intensity classifications, spatiotemporal continuity labels) and develop cross-modal consistency evaluation metrics (e.g., feature alignment accuracy, inter-sensor synchronization latency, semantic consistency scores). This research will enhance model generalization capabilities in complex urban traffic environments, establish standardized training benchmarks for smart city traffic management systems, and thereby accelerate the deployment of multi-sensor collaborative perception frameworks.

## Figures and Tables

**Table 1:** Effectiveness of different architectural designs (%).

Method	mAP	Rank-1
ResNet50	79.66	96.25
ResNet50-IBN	79.81	96.78
BoT50-ibn	80.10	96.72
ResNet50-IBN + BoT(IBNT-Net)	82.46	97.38

**Table 2:** Effectiveness of various optimizations on MHSA.

Method	mAP [%]	Rank-1 [%]	Params [M]	MACs [G]
MHSA	82.46	97.38	36.12	8.03
MHSA + reduction	82.53	97.38	—	—
MHSA + (FC + residual)	82.70	97.44	—	—
MHSA + reduction + (FC + residual)	83.03	97.56	35.44	7.85

**Table 3:** Results of different reductions of EMHSA (%).

Reduction Factor	mAP	Rank-1
8	82.44	97.62
16	83.03	97.56
32	82.70	97.56

**Table 4:** Effectiveness of multi-task learning optimization (%).

Method	MTL	mAP	Rank-1
ResNet50-IBN		79.81	96.78
	√	82.85	97.32
BoT50-IBN		80.10	96.72
	√	82.61	97.08
IBNT-Net		83.03	97.56
	√	84.85	97.68

**Table 5:** Result of different group convolution strategies.

Method	mAP [%]	Rank-1 [%]	Params [M]	MACs [G]
IBNT-Net	83.03	97.56	35.44	7.85
IBNT-DL4B	84.85	97.68	55.97	11.37
IBNT-DL2G	84.26	97.14	22.01	7.50
IBNT-DL4G	83.18	97.44	12.89	5.31

## Data Availability

The authors have used the publicly archived datasets VeRi-776 and VehicleID for this study.
